# Doctors in the making, or degrees for sale? A student's view of Brazil's medical education crisis

**DOI:** 10.3389/fmed.2025.1687117

**Published:** 2025-10-20

**Authors:** Vanessa M. S. Campos, Mateus Maggitti-Bezerril, Rodrigo C. Menezes, Luciana Sobral, Katia de Miranda Avena, Bruno B. Andrade

**Affiliations:** ^1^Instituto de Pesquisa Clínica e Translacional, Medicina Zarns, Clariens Educação, Salvador, Brazil; ^2^Multinational Organization Network Sponsoring Translational and Epidemiological Research (MONSTER) Initiative, Salvador, Brazil; ^3^Laboratório de Pesquisa Clínica e Translacional, Instituto Gonçalo Moniz, Fundação Oswaldo Cruz, Salvador, Brazil; ^4^Curso de Medicina, Escola Bahiana de Medicina e Saúde Pública, Salvador, Brazil; ^5^Department of Medicine, Division of Infectious Diseases, Johns Hopkins University, Baltimore, MD, United States; ^6^Department of International Health, Bloomberg School of Public Health, Johns Hopkins University, Baltimore, MD, United States

**Keywords:** medical education, private medical schools, residency training, healthcare workforce, Brazil

## 1 Introduction: historical background and expansion of medical schools

Medical education in Brazil dates back to the 19th century and was developed under strong European influence, characterized by a hospital-centered, technocratic model that primarily served the urban elite (1). Since its origins, medical training has been concentrated in major urban centers and traditional institutions, consolidating a centralized and selective system with limited engagement with the country's territorial and social diversity (2, 3).

As a direct consequence of the Brazilian Health Reform, the creation of the Unified Health System (SUS) in 1988 (4) introduced a new vision: to train physicians with a generalist profile, focused on primary care, territorially integrated, and socially committed. This profile, later incorporated into the National Curricular Guidelines for the Undergraduate Medicine Course (5), explicitly began to guide medical training in the country, establishing that graduates must act in an ethical, humanistic and socially responsible manner, with the ability to address the health needs of the population in different contexts and levels of care.

Beginning in the mid-2000s, Brazil experienced a steady expansion of undergraduate medical education. Acceleration occurred after 2013 with the enactment of the Ministry of Health's “Mais Médicos” Program (6–9). Between 2014 and 2024, dozens of new medical schools were authorized and thousands of new seats opened. A large portion of these new seats was allocated to private institutions, and many were added to existing programs rather than newly created ones (3, 10). This policy-driven growth, absorbed largely by the private sector, coincided with consolidation among major educational groups, uneven regional distribution, and constraints on infrastructure and supervised practice (3, 7, 11). These dynamics define the accelerating phase that frames our analysis in the following sections.

Drawing on our close observation of this transformation, we affirm that this vision has been increasingly challenged by an uncoordinated and accelerated expansion, evolving into a complex and fragmented process. It has been shaped by interiorization efforts, the surge of private institutions, the concentration of admissions in educational conglomerates, legal and political controversies, and a progressive decline in the competitiveness of admission processes (2). Paradoxically, this decline has unfolded alongside a marked increase in the pursuit of medical careers, a dynamic that has fueled the proliferation of new institutions while simultaneously eroding admission standards.

The landscape of medical education in Brazil thus presents unique challenges and opportunities capable of significantly shaping the academic and professional trajectories of future physicians. In this context, it is essential to cultivate a critical mass of students capable of analyzing, in a reflective and proactive manner, the reality in which they are embedded and considering its impact on professional practice. This opinion article offers an evidence-based analysis of the expansion of medical schools in Brazil, drawing on the authors' experience in medical education to describe the challenges faced, examine possible reforms, and highlight the forces that still drive the pursuit of excellence.

## 2 Expansion of medical schools and inequality in the distribution of medical school seats

As previously detailed in other publications, the acceleration of medical-school expansion after 2013 was catalyzed by the Ministry of Health's “Mais Médicos” Program (6, 8, 9), a health policy established by Law No. 12.871/2013 that integrates provision, training, and regulation. This policy context frames the analysis of how new seats and schools were distributed and how regional inequalities evolved during the 2014–2024 scale-up.

The quantitative expansion of medical schools in the country has not been accompanied by equivalent qualitative improvements, a fact evidenced by the Medical Demography in Brazil 2025 report (11). On the contrary, this growth triggered significant changes in the profile of students and faculty, raising concerns about the future of the medical profession. From our perspective, this is not merely a numerical issue, but rather a structural transformation whose social, ethical, and educational consequences remain unresolved and under-discussed.

In the past 10 years, the number of medical schools rose from 252 to 448, with nearly 28,000 new seats authorized (10). This is almost four times the number registered between 2004 and 2013. The turning point was the enactment of Law No. 12.871/2013, which created the “Mais Médicos” Program (6, 8, 9). Conceived as a Ministry of Health public policy, it integrates health and education dimensions through three axes: provision (deploying physicians to underserved and remote areas), training (expanding supervised practice opportunities), and regulation (criteria for authorizing new schools). While its aim was to mitigate regional inequalities, the policy's implementation was predominantly absorbed by the private sector and concentrated in already privileged regions (10), which limited its potential to achieve a more equitable distribution of physicians across the country.

**Figure 1:**
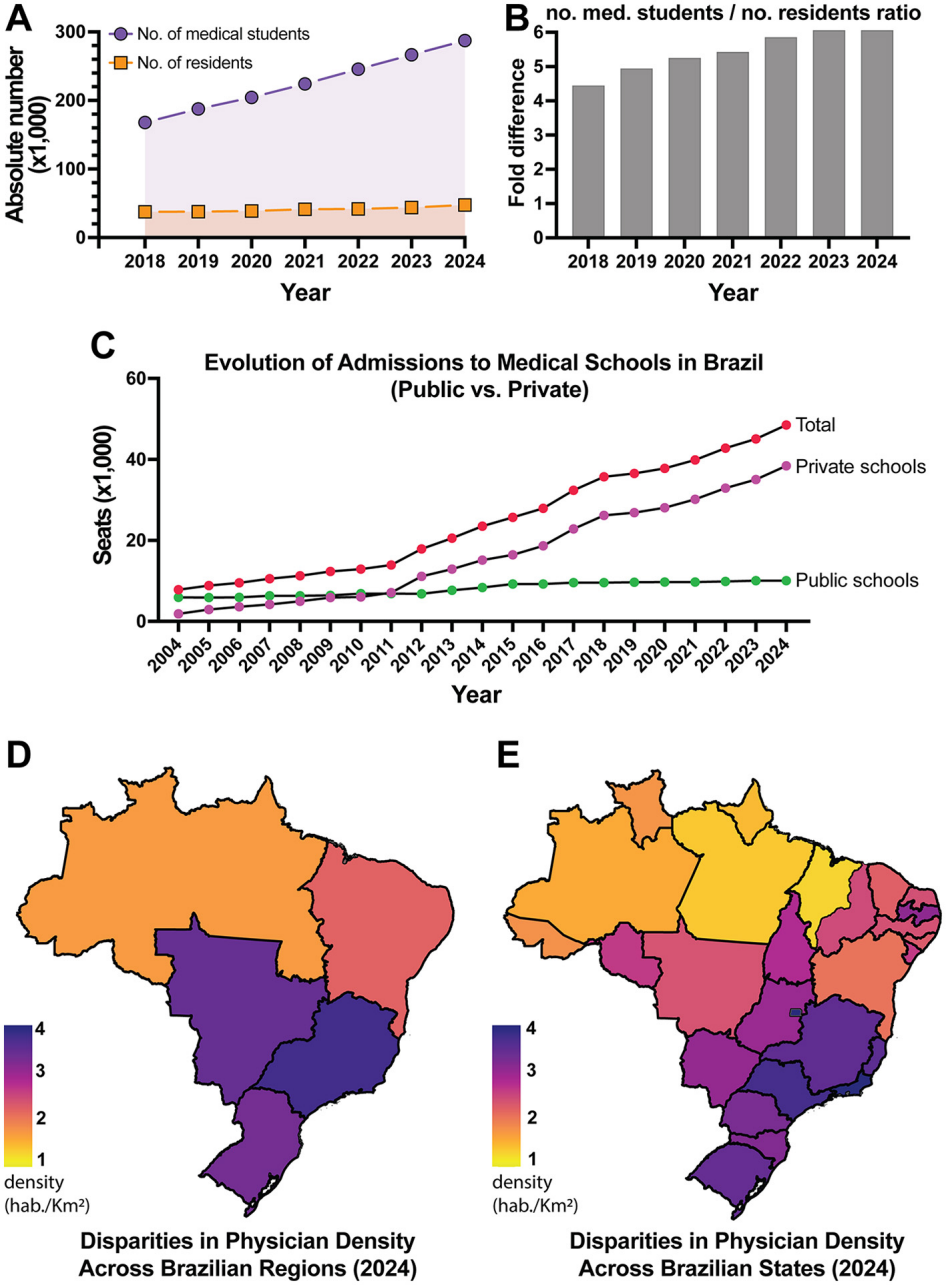
Trends and inequalities in medical education and physician distribution in Brazil. **(A)** Absolute number of medical students and residents in Brazil from 2018 to 2024, showing a consistent increase in both categories. The areas under the curves highlight the differences between annual totals. **(B)** Fold difference ratio between the number of medical students and residents over the same period, showing a widening gap over time. **(C)** Distribution of undergraduate medical seats by sector (public vs. private) and cumulative growth from 2010 to 2024, highlighting the growing predominance of the private sector. **(D)** Physician density per 1,000 inhabitants in Brazil in 2024, by region. **(E)** Physician density per 1,000 inhabitants in Brazil in 2024, by state. Data source: DIREM dashboard (https://produtos.direm.org/new-dashboard).

More than 91% of the new seats created since 2014 were allocated to private institutions, which by 2024 accounted for almost 80% of all undergraduate medical seats in the country (Figures 1A–C). This privatization of access has deepened territorial inequalities. While the Southeast region holds over 41% of seats, particularly in São Paulo and Minas Gerais, as a result, states such as Roraima, Acre, and Amapá account for just 1% of the national total (10, 11). It is evident that the so-called interiorization has occurred more on paper than in practice. Nearly 40% of the new seats were added to existing programs, often without proportional investment in infrastructure, supervision, or internship sites, as reported by Andrade et al. (10) and reinforced by the Federal Council of Medicine in its 2024 technical note highlighting deficiencies in newly accredited schools (12).

As a result, students face overcrowded classrooms, limited access to practical training, and weakened pedagogical follow-up. In addition to infrastructure constraints, rapid expansion increased class sizes and student-faculty ratios at many institutions (2, 10). National analyses during the expansion period report signs of academic decline, coinciding with the accelerated growth of for-profit schools, a pattern consistent with resource dilution and reduced opportunities for supervised practice (10). Although causality cannot be firmly established from observational data and from the authors' experience in medical education, warnings from the Federal Council of Medicine about insufficient infrastructure and supervision at newly accredited schools reinforce this concern (12, 13).

From a social equity standpoint, the predominance of private-sector seats shifts a substantial share of training costs to households through high tuition fees, increasing debt burdens and potentially narrowing access for lower-income and minority students (2, 14, 15). Given the spatial concentration of programs in wealthier regions, these financial barriers compound regional disparities in entry, persistence, and graduation (10, 11). Without targeted scholarships, social quotas, and student support services, expansion risks amplifying the very inequities it sought to reduce (14, 15).

From our perspective, this scenario is incompatible with the humanistic and socially responsive training required by the SUS model and advocated by the National Curricular Guidelines for the Undergraduate Medicine Course (5). This uneven expansion, predominantly led by private institutions, is graphically illustrated in the distribution of seats and their concentration across regions (Figures 1A–C). These patterns set the stage for understanding the persistent inequalities in the medical workforce, which will be discussed in the next section.

Publicly traded universities, in turn, have treated medical education as a financial asset (2). The commodification of the medical degree, once a symbol of public service and ethical responsibility, now serves market interests. In this context, training of future physicians risks becoming a business model rather than a societal mission.

These concerns are not isolated. In 2024, the Federal Council of Medicine (CFM) issued formal warnings regarding infrastructure deficiencies and supervision failures among rapidly growing institutions (12). We believe we are witnessing a crisis of meaning. What does it truly mean to train physicians today in Brazil? Are we producing professionals or merely degrees?

Rather than rejecting expansion altogether, we advocate for a more regulated, strategic, and socially aligned growth. The issue is not in the number of seats but in the lack of direction. A country with continental dimensions like Brazil cannot afford to neglect the quality, equity, and purpose of its medical education.

## 3 Profile and distribution of physicians in the post-expansion era

The expansion of medical education in Brazil has undoubtedly reshaped the demographic composition of the medical workforce. However, we argue that its practical impact on the healthcare system remains limited and, in many ways, problematic.

As of 2024, the Southeast region concentrated 55.5% of the country's physicians, while the North accounted for only 4.7% (11) (Figure 1D). These numbers reflect deep-rooted territorial inequalities that the simple increase in training vacancies has not been able to overcome. Although the national average stands at 2.81 physicians per 1,000 inhabitants, this figure masks stark regional disparities, as shown in the distribution of physicians across the national territory (Figures 1D, E). In Amazonas, for example, the ratio drops to 1.34, while the Federal District reaches 6.29 physicians per 1,000 inhabitants (11) (Figure 1E). These differences expose territorial inequities and challenge the notion that simply training more physicians will ensure broader access to care.

This imbalance is exacerbated by the growing influx of young physicians into the labor market. Professionals under the age of 34 now comprise about one-third of the active workforce (11). A large proportion are graduates of private institutions and tend to remain concentrated in major urban centers, where saturation is becoming increasingly evident. In parallel, the profession is undergoing a visible process of feminization (11). Women already constitute the majority among physicians aged 29 and under, accounting for 62.6% of this group (11). While these generational and gender shifts represent important changes in the profession's profile, they have not, by themselves, reduced gaps in underserved regions or improved working conditions for new physicians, which depend on structural policies and adequate incentives.

The sharp increase in the number of young professionals with limited clinical experience has revealed a set of structural problems documented in recent analyses. These include precarious employment and contractual instability, as highlighted in the Medical Demography in Brazil 2025 report (11), the saturation of opportunities in major urban centers (11), and the persistent shortage of medical residency positions relative to the number of graduates (16). Such conditions, as emphasized by Barbosa et al. (16), undermine the professional development of young physicians and expose systemic fragilities that cannot be overlooked. Taken together, these factors indicate a growing misalignment between the place of training, the place of practice, and the long-term prospects for integration into the public healthcare system.

Experiences from other Global South nations reveal similar challenges. In South Africa, for instance, the expansion of medical education has been accompanied by persistent inequalities in distribution, infrastructure, and retention of physicians in underserved areas (17). Comparable dynamics have also been described in Latin America. In Mexico, annual production of physicians outpaces the number of newly licensed specialists and persists alongside regional maldistribution (18). In Colombia, rapid growth in medical programs since the 1990s has raised concerns about quality and accreditation, while residency capacity remains limited (19–21). These patterns mirror global analyses showing that medical school proliferation can outpace the development of robust accreditation frameworks, with implications for equitable workforce integration (22).

Over the past two decades, Brazil has experienced a rapid expansion in medical education; however, growth in accredited residency positions and stable employment has not kept pace (2, 10). This imbalance has contributed to localized oversupply in major urban centers, underemployment, and concerns about declining returns to training, as the number of graduates increasingly exceeds the absorption capacity of the health system (11, 16). Here, market oversaturation denotes a structural mismatch in which the annual number of medical graduates outpaces the availability of residency positions and stable employment, producing local oversupply in large metropolitan areas despite national or regional shortages (23). In Brazil, this pattern is evident in the widening student-to-resident gap (Figure 1B) and in stark gradients in physician density per 1,000 inhabitants (Figures 1D, E), indicating urban saturation alongside peripheral scarcity (11). Similar dynamics have been reported in Mexico, where physician density coexists with regional maldistribution and bottlenecks in specialist training (18), and in Colombia, where rapid growth in medical programs has outpaced uniform accreditation and residency capacity (19–21).

If medicine follows this trajectory, the result could be a continuous production of graduates without ensuring their integration into the healthcare system in a qualified and equitable manner. This scenario risks not only the waste of human capital and public investment in education but also a deterioration in working conditions, the erosion of professional value, and the weakening of public healthcare delivery, ultimately undermining both the financial stability and the long-term career prospects of physicians.

## 4 Expansion and gaps in postgraduate training

Securing a spot in a medical residency has become one of the most formidable hurdles in the professional journey of newly graduated physicians. The number of graduates already exceeds the number of available positions, revealing a critical mismatch between the volume of medical training and the system's real capacity to absorb these professionals (11) (Figures 1A, B). This misalignment is not only evident in national statistics but has also been highlighted by the Pan American Health Organization, which estimates that 14 countries in the Americas, including Brazil, may face a shortage of up to two million healthcare workers by 2030 if structural reforms are not implemented (24).

Despite this projected deficit, training programs located in prestigious urban centers continue to attract intense competition, while strategic specialties such as family and community medicine, particularly in underserved areas, struggle to fill available positions, although it is an essential specialty within the SUS and has a growing number of authorized positions (25, 26). This imbalance reflects a structural disconnect between the type of specialists being trained and the country's actual healthcare demands.

In this scenario, many graduates, often burdened by substantial debt from private education, choose to enter the labor market immediately (2, 10). They commonly take on positions in emergency services or general practice, frequently without formal specialization (2, 10). This premature and sometimes underqualified entry into clinical care may compromise both the quality of service delivery and the professional development of these young physicians (27).

The fragility of residency programs further aggravates this situation. Residents often face exhausting work schedules, low stipends with limited purchasing power, inadequate infrastructure, and insufficient supervision (11, 16, 27). These adverse conditions compromise not only the quality of the training process but also the wellbeing and motivation of future specialists, creating a fertile ground for burnout and other mental health challenges that can have lasting effects on their professional trajectories.

A recent policy proposal has added another layer of concern. In April 2025, the Brazilian Ministry of Education launched the National Examination for the Assessment of Medical Education (ENAMED) (28), designed as a nationwide exam to evaluate graduating medical students. Closely linked to the National Medical Residency Examination (ENARE) (29), the ENAMED serves as the standardized test whose scores are used to compete for residency positions through the ENARE system. By disregarding students' broader academic records, including engagement in research, teaching activities, and community outreach, this measure undermines the meritocratic value of the educational journey and narrows the evaluation process to standardized test performance, diminishing the recognition of diverse academic trajectories. Potential benefits of a national exam depend on fair, transparent design and broad competency coverage; narrow testing formats risk overlooking essential dimensions of training.

In our view, this reform reinforces an overly utilitarian model of medical education. In a system already marked by inequalities and lapses in quality, removing one of the few mechanisms that still valued comprehensive and socially engaged training pushes the formation of physicians further away from the ethical and humanistic principles that should underpin healthcare in Brazil.

At the transition to practice, the cumulative costs of pursuing residency (application fees, examination preparation, relocation, and foregone earnings) can be prohibitive for some graduates, contributing to delayed entry or diversion to non-specialist tracks (10, 16). These financial barriers interact with saturated urban markets and fragmented contracts, shaping early-career trajectories (11). Signals of emigration intent among new physicians have been noted anecdotally in professional forums, but robust national estimates remain limited (23).

## 5 Shortcuts to specialization: lato sensu as a symptom, not a solution

Lato sensu postgraduate programs, a category specific to the Brazilian system of non-degree specialization courses, typically lasting 1 to 2 years, offered by private institutions, providing targeted professional training, and not granting the official specialist title recognized by the National Medical Residency Commission, have been gaining popularity among newly graduated physicians. For a broader discussion of postgraduate tracks for medical graduates in Brazil, see (2). These courses have been considered an alternative in the face of the shortage of medical residency positions, which represent the formal and accredited pathway to professional specialization in the country.

While these programs may offer certifications and opportunities for focused study in specific areas, they frequently lack the technical rigor, structured supervision, and clinical immersion that define accredited residency training. This limitation not only results in a fragmented and insufficient educational experience but also risks producing professionals with uneven competencies, a concern that is particularly critical in fields where intensive hands-on practice is indispensable for safe and effective patient care.

In an official statement, the Federal Council of Medicine reaffirmed that lato sensu programs do not confer the title of medical specialist (30). This clarification weakens the legitimacy of such certifications in the job market and introduces a layer of uncertainty into the professional trajectory of young graduates. In many cases, the adoption of this path is less a matter of free choice and more a reflection of systemic failure. By shifting responsibility for structural shortcomings to the individual, the system leaves physicians to navigate a fragmented and torn landscape with little institutional support.

This trend, when combined with the financialization of medical education and the lack of a unified national strategy for medical specialization, reinforces educational inequalities and undermines any coherent commitment to the delivery of quality care. The consequences extend beyond personal career insecurity and translate into systemic threats to the equity, safety, and effectiveness of healthcare provision.

Faced with this reality, the future of medical education in Brazil appears marked by contradictions. The numeric expansion of schools and training slots, in the absence of coordinated planning, compromises the quality of formation and jeopardizes the sustainability of a healthcare system that urgently requires professionals who are both technically competent and socially responsive to the country's deep regional disparities.

The widening gap between training and labor market demands, coupled with the precarious state of residency programs and the rise of insufficient alternatives for specialization, points toward a scenario of professional oversaturation, clinical vulnerability, and growing disillusionment among new physicians. Brazil runs the risk of producing an ever-increasing number of medical graduates without providing the conditions needed to train specialists committed to excellence and equity in healthcare.

In this context, we believe medical education must be elevated to the status of a strategic national priority. Without collective commitment, long-term vision, and policy coherence, the likely outcome is the consolidation of a model that multiplies diplomas while progressively emptying them of their meaning.

## 6 Opportunities and drivers of excellence

Despite these structural challenges, there are still glimpses of opportunity that deserve attention and critical investment. Initiatives such as the “Mais Médicos” Program (6, 8, 9) have attempted to reduce healthcare disparities by incentivizing physicians to practice in underserved regions and creating supervised primary-care settings that indirectly support training. While the program has faced criticism and political resistance, it has nonetheless provided valuable clinical experience and, in many cases, a sense of professional purpose and stability for young doctors entering the workforce (9).

The growing recognition of competency-based education and the implementation of national licensing exams may also contribute to standardizing the quality of medical training. By establishing a minimum threshold of competency, these measures can help ensure that graduates, regardless of their institution of origin, possess essential clinical skills and ethical foundations.

International collaborations and student exchange programs are beginning to expand the horizons of medical education. These experiences expose students to different healthcare systems, promote cross-cultural learning, and offer access to more advanced training opportunities. Although still limited to a small segment of the student population, such initiatives represent a positive shift toward a more global and evidence-based medical education model (31–33).

Additionally, some institutions have started to prioritize research and foster environments that encourage scientific inquiry. This movement, although still incipient in many contexts, is essential to strengthening the foundation of evidence-based practice and nurturing a generation of physicians capable of both critical thinking and innovation.

## 7 The future medical student: a vision

The profile of the future medical student will be shaped by ongoing transformations in clinical practice, medical education, and societal demands. In an increasingly saturated job market and within a medical field marked by rising complexity, standing out will require more than academic excellence alone. Future physicians will need to combine technical mastery with adaptability, critical thinking, communication skills, and familiarity with emerging technologies such as artificial intelligence and data analytics (27, 34).

These changes are redefining the very concept of medical competence. Knowledge of bioinformatics, digital ethics, global health, and healthcare management is becoming essential to address contemporary challenges. At the same time, humanistic attributes such as empathy, active listening, and a sense of purpose will remain fundamental, reaffirming the importance of compassionate care in an era of growing automation (34, 35).

The context in which this new professional profile will emerge is far from neutral. According to projections from Medical Demography in Brazil 2025 (11), the country may reach one million active physicians by 2035. Although this growth is significant, it carries considerable implications for the labor market. It intensifies competition in major urban centers, exacerbates the gap between medical education and postgraduate specialization, and compels future professionals to develop strategies for differentiation within a rapidly expanding workforce.

Soon, becoming a physician will require the ability to navigate confidently across diverse domains, from a strategic understanding of the labor market to technological innovation and, above all, a steadfast commitment to patient-centered care.

## 8 A call for reform

For medical education in Brazil to contribute meaningfully to a sustainable healthcare future, reforms must prioritize quality over quantity. It is essential to establish stricter criteria for the authorization of new medical schools, invest in robust faculty development programs, and ensure the effective integration of students into public health networks from the early stages of their training. These measures would help align educational structures with the real demands of the healthcare system and reduce the current mismatch between formation and professional practice.

Expanding the number of residency positions and offering financial incentives for graduates who choose to specialize in critical areas, particularly those neglected by market dynamics, represent concrete strategies to address regional imbalances in physician distribution. Such policies must be grounded in long-term planning and a commitment to social equity, rather than short-term solutions driven by institutional interests.

Ultimately, we believe that with coherent public policies and a collective commitment to improving the medical education system, it is possible to form professionals who are not only technically competent but also compassionate and socially engaged. Although the path forward remains challenging, the continued dedication of students and educators, along with a renewed vision for the role of medicine in society, offers a foundation for progress. This viewpoint, grounded in the authors' experience in medical education and observation of trainee trajectories, seeks to contribute to the broader debate on how medical education in Brazil can reconcile expansion with quality, equity, and purpose.

## 9 Conclusion

In sum, grounded in the authors' experience in medical education and the evidence reviewed, Brazil's rapid scale-up of medical schools has exposed critical bottlenecks in the distribution of physicians, supervised practice, and postgraduate training. Aligning authorization and accreditation with capacity in clinical training and residency, financing equity-oriented student support, and deploying distribution and retention incentives aligned with SUS needs are immediate priorities. With coherent, data-driven policies, expansion can be translated into gains in quality, equity, and sustainable professional development, rather than localized oversupply and systemic strain.
